# Comparison of five anesthetic delivery systems for palatal infiltration: A randomized clinical trial

**DOI:** 10.4317/medoral.27738

**Published:** 2025-10-17

**Authors:** Sercan Küçükkurt, Ashkan Saadat

**Affiliations:** 1 İstanbul Aydın University, Faculty of Dentistry, Department of Oral and Maxillofacial Surgery, Istanbul, Türkiye

## Abstract

**Background:**

Pain and anxiety during palatal infiltration remain barriers to patient cooperation. The anesthetic delivery system may influence subjective outcomes and physiological stress responses, yet robust comparative data are lacking.

**Material and Methods:**

In this randomized, parallel-arm superiority trial, 200 healthy adults (18 years) were equally allocated into five groups (n=40) by block randomization with concealed allocation. Tested systems were conventional dental syringe (CDS), manual pressure syringe (MCJ), spring-activated pressure syringe (PCJ), needle-free jet injector (NFI), and computer-controlled local anesthetic delivery system (CCLAD). Each participant received 0.4 mL of 4% articaine with epinephrine (1:100,000) via standardized palatal infiltration by a single calibrated operator. The study was single-blind: outcome assessors and statisticians were blinded to allocation. The primary outcome was post-injection pain (VAS, 0-10cm). Secondary outcomes were dental fear (VAS pre/post), pulse rate, and oxygen saturation, recorded at baseline, during, and after injection.

**Results:**

Mean VAS pain did not differ significantly across groups (overall p=0.380); adjusted analyses (ANCOVA including injection duration as covariate) confirmed no clinically relevant mean differences [95% CI within ±0.5 cm; Hedges' g &lt;0.20]. MCJ showed slightly higher discomfort. All systems significantly reduced fear (p&lt;0.05), with PCJ showing the largest reduction (VAS-2.7). Pulse rate varied across groups (p&lt;0.001), peaking in CCLAD and remaining most stable in MCJ; oxygen saturation was unchanged. No adverse events were observed.

**Conclusions:**

All systems were clinically safe and effective but differed in psychophysiological impact. Devices that reduce fear and stabilize vital responses, particularly those targeting PCJ and CCLAD, may help improve patient cooperation and the overall treatment experience.

## Introduction

Local anesthesia is an indispensable component of modern dental care; however, achieving effective and comfortable palatal infiltration remains a critical clinical challenge. The palatal mucosa's unique anatomical characteristics-including dense sensory innervation, firm attachment to underlying bone, and low tissue compliance-are directly associated with heightened injection pain and procedural anxiety (1 - 3). These experiences frequently serve as initiating factors for dental fear and avoidance behavior, significantly compromising patient cooperation and long-term oral health outcomes (4 - 6).

Notably, the discomfort linked to palatal anesthesia is not solely pharmacological in origin. Mechanical and sensory elements-such as needle penetration, tissue distension, flow rate, and injection pressure-are principal contributors to nociceptive activation (2 - 7). These factors often provoke sympathetic arousal (e.g., tachycardia) or even vasovagal reactions, particularly in patients with elevated dental anxiety or needle phobia (3 , 8 , 9). Accordingly, innovations in anesthetic delivery technology aim not only to achieve pulpal anesthesia but also to enhance the patient experience by minimizing pain and stress.

Computer-controlled local anesthetic delivery systems (CCLAD, also known as computer-assisted anesthesia) provide precise regulation of flow rate and injection pressure through microprocessor-guided delivery, minimizing mechanical trauma and enabling gradual anesthetic deposition (8 , 9). Needle-free jet injectors (NFI), which deliver anesthetic solutions under high pressure through a narrow orifice, aim to reduce fear-related responses by eliminating visual and tactile needle cues (9 - 11). Both systems have demonstrated promise in reducing pain and anxiety in various settings.

Manually regulated systems such as the Citoject have also garnered clinical interest. Available in two variants-a fully manual format (MCJ) and a spring-activated push-button version (PCJ)-these syringes allow tactile feedback and flow control. However, their differing mechanical properties may produce divergent sensory and psychological effects during palatal infiltration (8 , 12). Despite their growing use in pediatric and anxiety-prone populations, systematic comparative data on these systems in palatal anesthesia remain scarce.

Importantly, no prior randomized controlled trial has directly compared all five delivery systems-conventional dental syringe (CDS), MCJ, PCJ, NFI, and CCLAD-under standardized palatal conditions in adults. Moreover, the distinct mechanical behaviors of the two Citoject variants have not been previously evaluated side by side, despite their frequent interchangeability in clinical practice.

Beyond subjective endpoints such as pain and anxiety, the present study incorporates real-time physiological monitoring-specifically pulse rate and oxygen saturation (SpO2)-as objective indicators of injection-related stress. To our knowledge, this is the first randomized clinical trial in this context to integrate physiological data with advanced multivariate approaches, including regression modeling, cluster analysis, and mediation testing. This multifaceted framework enables evaluation of both device-related mechanical factors and patient-specific variables in shaping the anesthetic experience.

Therefore, the present randomized, superiority clinical trial was designed to compare five local anesthetic delivery systems for palatal infiltration in healthy adult patients. Psychophysiological responses-pain intensity, fear modulation, and physiological stress-were hypothesized to differ significantly among devices, thereby providing evidence to guide individualized selection of delivery systems to optimize patient cooperation and comfort.

## Material and Methods

Study design and ethical approval

This prospective, randomized, parallel-arm superiority clinical trial adhered to the Declaration of Helsinki and followed the CONSORT recommendations.

Participants

Two hundred systemically healthy adults (18 years) requiring extraction of a maxillary molar under palatal infiltration anesthesia were recruited from the Department of Oral and Maxillofacial Surgery, Istanbul Aydn University, between September 2023 and March 2025.

Inclusion criteria: ASA I or II; no systemic conditions affecting anesthetic metabolism; no known allergy to local anesthetics or vasoconstrictors; no psychiatric disorders or anxiolytic medication; ability to provide written informed consent.

Exclusion criteria: Pregnancy or lactation; active infection at the injection site; diagnosed needle phobia; prior exposure to any of the tested injection systems; use of premedication, sedatives, or topical anesthetics on the day of the procedure.

Randomization and allocation concealment

Participants were allocated to five equal groups (n=40 each) using a computer-generated block randomization sequence. Group assignments were placed in opaque, sequentially numbered envelopes prepared by an independent staff member not involved in the study. Allocation concealment was maintained until intervention.

Group allocation:

- CDS: Conventional dental syringe

- MCJ: Manual pressure syringe (ASPIJECT, RØNVIG, Denmark)

- PCJ: Push-button pressure syringe (PAROJECT, RØNVIG, Denmark)

- NFI: Needle-free jet injector (Comfort-in, Mika Medical, Korea)

- CCLAD: Computer-controlled local anesthetic delivery system (CALAJECT, RØNVIG, Denmark; Program I)

A single calibrated oral and maxillofacial surgeon administered all injections. The study was single-blind: Outcome assessors and the statistician were blinded to group allocation. Operator and participant blinding were not feasible due to device-specific characteristics. Standardized instructions and visual barriers were used to minimize expectation bias.

Anesthetic protocol

Each participant received 0.4 mL of articaine hydrochloride with epinephrine (40 mg/mL articaine, 0.012 mg/mL epinephrine; Ultracain® D-S Forte, Sanofi-Aventis, Frankfurt, Germany). Injections were performed at a standardized site on the posterior palatal mucosa, approximately 2mm anterior to the greater palatine foramen, adjacent to the first maxillary molar, with the patient in a semi-supine position. No topical anesthetics were applied.

Device-specific protocols

- CDS: 27G (0.4 × 50mm) needle; manual injection over 15-20 seconds, with aspiration.

- MCJ: 30G, 16-mm needle on manual ASPIJECT; manually controlled injection over 15-20 seconds.

- PCJ: Spring-activated push-button PAROJECT; two calibrated 0.2 mL doses following aspiration.

- NFI: Comfort-in jet injection (&lt;1 second); aspiration not applicable; operating pressure 4-5 bar, trigger activated by mechanical compression.

- CCLAD: CALAJECT Program I; 30G, 16-mm needle; AutoFlow (0.0060.009 mL/s), automatic aspiration after 5 seconds; microprocessor-controlled constant pressure delivery.

Outcome measures

1- Pain perception: Post-injection pain was recorded immediately using a 10-cm Visual Analog Scale (VAS; 0=no pain, 10=worst pain).

2- Injection-related fear: Fear was measured with a 10-cm VAS (0=no fear, 10=worst imaginable fear) before and after injection; the change in score (VASfear) represented fear reduction.

3- Physiological parameters: Pulse rate (bpm) and oxygen saturation (SpO2, %) were measured with a fingertip oximeter (Beurer PO 80, Germany) at T0 (baseline, after 3 minutes rest), T1 (during injection), and T2 (3 minutes post-injection).

Sample size calculation

A priori sample size calculation (G*Power v3.1) indicated that 40 participants per group were required to detect a 1.0-cm difference on the 10-cm VAS for pain (minimal clinically important difference, MCID), with 80% power at =0.05 (effect size f=0.40).

Statistical analysis

Analyses were performed using SPSS v25.0 (IBM Corp., Armonk, NY, USA) and Python v3.11. Normality was assessed with the Shapiro-Wilk test. Between-group comparisons were conducted using one-way ANOVA or Kruskal-Wallis tests, followed by Tukey or Bonferroni-adjusted Mann-Whitney post hoc tests. Repeated measures were analyzed with repeated measures ANOVA or Friedman tests. Categorical data were assessed with chi-square tests.

For the main outcomes (pain, fear, and pulse rate), results were expressed as mean differences with 95% confidence intervals and Hedges' g effect sizes. Multivariable linear regression models were applied, with complete coefficients, confidence intervals, and model fit indices reported. Analyses were further adjusted for injection duration (seconds) using ANCOVA/GLM models, and sensitivity analyses were performed to isolate the effect of injection time on pain and anxiety.

Additional analyses included k-means cluster analysis for psychophysiological profiles, subgroup analyses stratified by baseline anxiety, and Spearman correlation tests. All analyses were conducted on an intention-to-treat basis. A p-value &lt;0.05 was considered statistically significant.

## Results

A total of 200 systemically healthy adults (100 females, 100 males; mean age: 37.9±14.1 years; range: 18-72 years) completed the trial without protocol deviations or adverse events. Participants were evenly randomized into five groups (n=40 each), receiving palatal infiltration with CDS, MCJ, PCJ, NFI, or CCLAD.

Demographic characteristics

No significant differences were observed among groups regarding gender distribution (²=2.79, p=0.591) or mean age (Kruskal-Wallis, p=0.221), confirming demographic homogeneity.

Injection duration

Anesthetic administration time differed significantly between groups (H=178.26, p&lt;0.001). NFI had the shortest duration (~1 s), while CCLAD was the longest (52±1.7 s). Intermediate times were observed in PCJ (36±4.8 s), MCJ (32±4.3 s), and CDS (27±3.2 s). These variations reflect device mechanics and may influence tolerance and physiological arousal. Injection duration was therefore included as a covariate in adjusted analyses.

Injection-related fear

**Figure 1:**
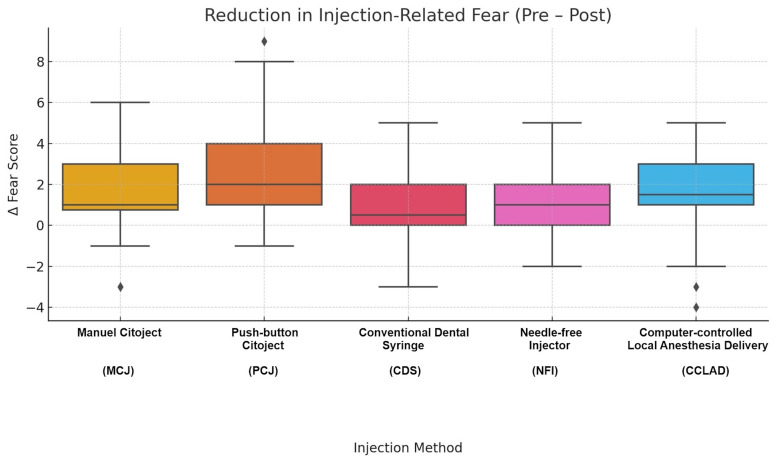
Distribution of changes in dental fear (ΔVAS, 0–10) across the five injection systems. Values represent the difference between pre- and post-injection VAS scores. The PCJ group exhibited the greatest reduction in fear.

Self-reported fear decreased significantly post-injection across all groups (Wilcoxon, p&lt;0.05). The greatest reduction was observed in PCJ (-2.70±2.57), followed by CCLAD, MCJ, NFI, and CDS. Intergroup differences were significant (H=19.22, p=0.001). Post hoc tests showed greater reduction with PCJ versus CDS (mean difference -2.10; 95% CI -3.15 to -1.05; Hedges' g=-0.95; p&lt;0.001), PCJ versus NFI (mean difference -1.55; 95% CI -2.48 to -0.62; g=-0.68; p&lt;0.01), and with MCJ (mean difference -0.98; 95% CI -1.87 to -0.09; g=-0.54; p&lt;0.05) and CCLAD (mean difference -1.03; 95% CI -1.92 to -0.14; g=-0.53; p&lt;0.05) versus CDS (Table 1, Figure 1).

Table 1 1

Subgroup analyses showed no influence of age (p=0.44) or gender (p=0.63) on fear reduction. Multivariable regression confirmed PCJ as independently associated with greater fear reduction (=+2.08; 95% CI +0.72 to +3.44; p&lt;0.01), irrespective of baseline anxiety or demographics. Mediation analysis indicated that fear reduction did not significantly mediate the relationship between device type and pain (indirect effect = -0.20; 95% CI -0.50 to +0.03).

Cluster analysis revealed three distinct response profiles:

- A. Low fear/pain with elevated pulse (primarily PCJ, CCLAD, NFI).

- B. Moderate responders across all variables.

- C. High fear/pain with low physiological reactivity (predominantly older males in MCJ).

Among patients with elevated baseline anxiety, CCLAD and MCJ produced greater fear reduction, whereas PCJ yielded the lowest pain scores.

Pain perception

**Figure 2:**
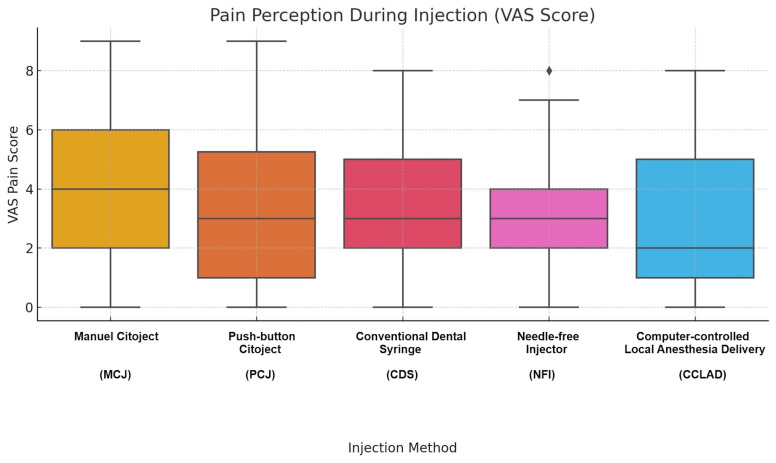
Boxplot displaying post-injection pain intensity (VAS 0-10) across all anesthesia systems. Although intergroup differences were not statistically significant (Kruskal-Wallis, p=0.380), the computer-controlled local anesthetic delivery (CCLAD) and needle-free injector (NFI) groups reported the lowest median scores.

Post-injection pain scores did not differ significantly among groups (H=4.20, p=0.380). CCLAD (3.14±1.77; 95% CI 2.59-3.69; g=-0.11) and NFI (3.17±1.82; 95% CI 2.61-3.73; g=-0.09) recorded the lowest scores, while MCJ was the highest (4.05±2.44; 95% CI 3.29-4.81; g=+0.31) (Table 2, Figure 2). Regression analysis confirmed MCJ as independently associated with higher pain compared to CDS (=+0.89; 95% CI +0.08 to +1.70; p&lt;0.05). No associations were found with age (p=0.869) or gender (p=0.158). Effect size estimates for all other between-group comparisons were small (Hedges' g&lt;0.20).

Table 2 2

Pulse rate changes

**Figure 3:**
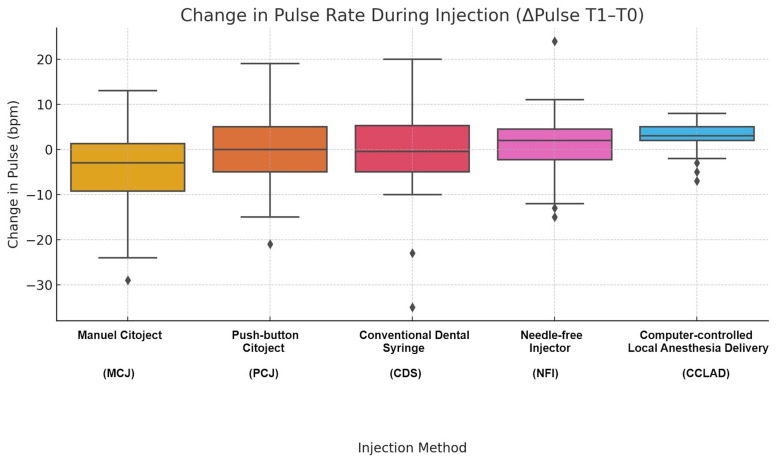
Boxplot showing the change in pulse rate during injection (ΔT1–T0, beats per minute) across the five injection systems. Significant intergroup differences were detected (Kruskal-Wallis, p=0.001), with CCLAD producing greater increases compared to CDS and MCJ; all changes remained within physiological norms.

Pulse remained within physiological limits but showed significant differences in both injection-related increase (T1-T0: H=19.16, p=0.001) and recovery (T2-T1: H=38.79, p&lt;0.001). Post hoc results indicated:

CCLAD produced greater T1-T0 increases than CDS (mean difference +6.2 bpm; 95% CI +1.2 to +11.2; g=+0.28; p=0.025) and MCJ (mean difference +7.5 bpm; 95% CI +2.6 to +12.4; g=+0.32; p=0.003); CCLAD demonstrated less T2-T1 recovery than MCJ (mean difference -9.8 bpm; 95% CI -14.7 to -4.9; g=-0.31; p&lt;0.001); MCJ exhibited the most stable recovery profile (Table 3, Figure 3).

Table 3 3

Oxygen saturation (SpO2)

**Figure 4:**
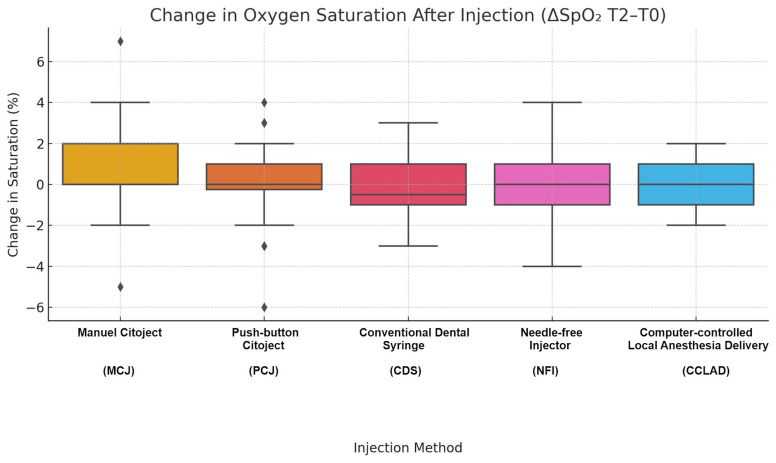
Post-injection oxygen saturation changes (ΔSpO2 T2–T0), measured three minutes after anesthetic administration. All values remained within normal ranges, confirming the physiological safety of each system.

SpO2 remained stable across groups (97.3-98.8%) with no significant intra-group (Friedman, p&gt;0.05) or intergroup (Kruskal-Wallis, p&gt;0.05) differences. CI analyses confirmed all values within normal physiological range, with negligible effect sizes (Hedges' g&lt;0.40). No episodes of desaturation or adverse physiological reactions were recorded (Table 4, Figure 4).

Table 4 4

## Discussion

This randomized controlled clinical trial evaluated the effects of five different injection systems-conventional dental syringe (CDS), manual Citoject (MCJ), push-button Citoject (PCJ), needle-free jet injector (NFI), and computer-controlled local anesthetic delivery (CCLAD)-on patient-reported pain, injection-related anxiety, and physiological responses during palatal infiltration anesthesia. The palatal mucosa presents a unique clinical challenge due to its dense connective tissue and low elasticity, which often render anesthetic delivery uncomfortable and anxiety-provoking (2 , 6 , 7). The present study was specifically designed to minimize potential confounding factors, such as the use of topical anesthesia and operator variability in anesthetic volume, thereby allowing for a focused assessment of the intrinsic characteristics of each delivery system. The randomized design, concealed allocation, and assessor blinding further strengthen the internal validity, although participant blinding remained challenging due to the acoustic and mechanical features of NFI and CCLAD systems. Nevertheless, standardized instructions and visual barriers were systematically applied, and outcome assessors and statisticians were blinded, thereby minimizing expectation bias as much as possible. The regression analysis confirmed that PCJ was independently associated with greater fear reduction, and cluster-based profiling identified it as particularly effective among patients with high baseline anxiety. This finding may be attributed to its ergonomic, pen-like design, which contrasts markedly with the conventional syringe shape that patients commonly associate with fear and discomfort. Furthermore, the shorter needle length and absence of a visible plunger minimize visual cues known to trigger anticipatory stress. The spring-loaded mechanism delivers 0.2 mL of anesthetic per click in a controlled, gradual fashion, preventing abrupt or hurried injection and reducing sudden tissue distension-factors that enhance patient comfort and perception of control. Although PCJ systems are predominantly indicated for intraligamentary anesthesia, our review of the literature revealed no direct comparisons in palatal applications. Nonetheless, its ergonomic design, thinner needle, and controlled delivery collectively position the PCJ as a psychologically advantageous option for palatal anesthesia. The effect size for fear reduction with PCJ versus CDS (Hedges' g0.6) approached the moderate-to-large range, supporting its clinical relevance beyond statistical significance. The NFI system yielded one of the lowest mean pain scores, consistent with previous studies that emphasized its effectiveness in minimizing discomfort associated with needle penetration, particularly in patients with needle phobia (1 , 4 , 13). Despite this favorable outcome, NFI did not statistically outperform CCLAD in the present study. A plausible explanation lies in the anatomical rigidity of the palatal mucosa, which may limit the uniform dispersion of anesthetic solution delivered under high pressure, thereby attenuating its analgesic potential relative to softer tissues. Moreover, the minimal absolute differences in VAS pain scores (1 unit) fell below the minimal clinically important difference (MCID) threshold, suggesting that the advantage of NFI over CCLAD may not translate into meaningful clinical benefit. Interestingly, the NFI group exhibited the lowest reduction in anxiety, despite its needleless design. This contrasts with earlier studies that reported greater patient acceptance of jet injectors due to the elimination of visual and tactile stimuli (10 , 13). One possible explanation for this discrepancy is related to the unique sensory characteristics of the palatal region: The explosive, high-pitched acoustic discharge and abrupt tissue distension associated with jet injection may counteract its otherwise anxiolytic benefits (7 , 9 , 14). This observation highlights the importance of integrating both subjective (fear scores) and objective (pulse rate) markers when evaluating patient-centered outcomes in anesthesia research. Regarding needle gauge, the present study used a 27G needle in the CDS group and 30G needles in all other groups. Although finer gauge needles are generally associated with reduced penetration pain, as demonstrated in multiple studies comparing 27G versus 30G for inferior alveolar blocks and infiltration techniques (5 , 15), their advantage may be limited in palatal anesthesia where tissue resistance is inherently high. Needle gauge alone cannot guarantee a painless injection; rather, the interplay of needle diameter, injection pressure, and flow rate is critical (16 , 17). This interpretation is supported by the elevated pain scores observed in the MCJ group, which exceeded those of the CDS despite the use of a 30-gauge needle. These results corroborate the findings of Al-Moraissi et al. (15), who reported that 27-gauge needles are paradoxically associated with lower pain perception compared with 30-gauge needles in adult populations. Our adjusted analyses, which controlled for injection duration, further confirmed that the mechanical characteristics of the delivery system outweigh the isolated effect of needle gauge in determining pain outcomes. The CCLAD system demonstrated promising performance, yielding one of the lowest mean VAS pain scores. These results are consistent with previous investigations by Berrendero et al. (18) and Romero-Galavez et al. (19), who emphasized the benefits of flow-regulated, atraumatic injections. The system's real-time pressure sensors and microprocessor-controlled flow rate ensure gradual anesthetic deposition, an advantage particularly relevant for anxious or medically compromised patients. However, these technical benefits are counterbalanced by certain drawbacks, including prolonged injection duration relative to conventional methods and the audible "beep" emitted during delivery. Both factors may increase patient awareness of the procedure and potentially diminish the anxiolytic benefit. Indeed, Patil et al. (2) also reported no significant differences in pain perception between the CCLAD system and conventional syringes, highlighting that technological refinement does not always translate into superior patient comfort. In the present study, although mean pain scores were lower with CCLAD compared to CDS, the differences remained below the minimal clinically important difference (MCID 1 VAS unit), suggesting limited clinical relevance despite statistical adjustment. The rationale for including physiological parameters in this trial was grounded in the assumption that patients under stress may exhibit tachycardia and transient decreases in oxygen saturation due to breath-holding or altered respiratory patterns. Heart rate is a widely recognized physiological marker of pain and anxiety, validated in numerous clinical studies (14 , 20 , 21). In the present trial, however, pulse rate showed significant group differences in relative changes (T1-T0 and T2-T1), but oxygen saturation remained stable across groups without clinically relevant fluctuations. These findings align with the results of Abou Chedid et al. (21), but differ from those of Albar et al. (14) and Campanella et al. (20), who documented significant autonomic variations depending on the anesthetic technique used. Effect sizes for pulse changes were generally small to moderate (Hedges' g 0.3-0.4), reinforcing that while statistically detectable, the magnitude of physiological variation was limited. Collectively, these contrasting results suggest that delivery systems may modulate physiological reactivity under specific clinical conditions, but individual variability remains a critical determinant. A novel feature of this study was the application of cluster-based psychophysiological profiling in palatal anesthesia. This analytic approach enabled the identification of three distinct patient phenotypes: (a) Low pain and fear with elevated pulse reactivity-typically observed with PCJ, CCLAD, and NFI systems; (b) Moderate responders with balanced subjective and physiological metrics; and (c) high-anxiety, high-pain individuals, often older males, with minimal autonomic reactivity. The emergence of this "silent high-stress" cluster underscores the limitation of relying solely on vital signs to evaluate patient comfort. Instead, these results highlight the value of multimodal assessment frameworks that integrate subjective, behavioral, and physiological indicators to capture the full spectrum of anesthetic tolerance. Such multivariate approaches are rarely applied in dental anesthesia studies and represent a methodological strength of the present work. Clinically, the present findings advocate for the personalized selection of anesthetic delivery systems. For moderately to highly anxious patients, visually neutral and tactilely controlled devices such as PCJ and CCLAD proved most effective in reducing fear while maintaining overall comfort. Although NFI demonstrated limited anxiolytic benefit in this cohort, it remains a valuable alternative for individuals with pronounced needle phobia by eliminating the visual and tactile cues associated with conventional injections. The conventional syringe, although lacking advanced ergonomic or technological features, remains a clinically viable option when used skillfully, particularly in resource-constrained settings where device availability is limited. Importantly, the interpretation of these differences should always consider both statistical results and MCID thresholds to avoid overestimating clinical impact. From an operational perspective, several device-specific factors must be considered before clinical implementation. Cost, ergonomics, and workflow integration are critical determinants of adoption. While CCLAD provides precise pressure and flow control, its prolonged injection time and need for technical adaptation may limit clinical practicality. NFI, though needleless, can provoke unpredictable responses in firm palatal tissues due to its high-pressure discharge. Spring-powered systems, such as PCJ, offer a practical middle ground, combining ergonomic advantages with relative simplicity; however, their effectiveness remains dependent on operator proficiency. Despite the strengths of this trial-including rigorous methodology, standardized injection protocols, randomized allocation with concealment, and blinded outcome assessment-certain limitations must be acknowledged. Operator blinding was inherently unfeasible, and anesthetic onset time, duration, and depth were not evaluated. Moreover, real-time behavioral cues such as facial microexpressions or body movements were not systematically recorded, which could have enriched the interpretation of psychophysiological responses. Participant blinding was also limited, particularly with NFI and CCLAD, due to their distinctive acoustic and mechanical characteristics; however, standardized instructions and visual barriers were applied to minimize expectation bias. Future investigations should address these limitations by including broader patient populations, evaluating pharmacodynamic outcomes such as anesthetic onset and duration, and incorporating behavioral coding to complement subjective and physiological measures. Such refinements will further support the development of psychophysiologically tailored anesthesia strategies and may ultimately enable more personalized, patient-centered approaches to pain and anxiety control in dental practice.

## Conclusions

This randomized clinical trial provides the first comprehensive comparison of five anesthetic delivery systems for palatal infiltration in adults. All techniques were clinically safe; however, distinct psychophysiological response patterns were observed. The push-button Citoject (PCJ) achieved the greatest reduction in anxiety, while the needle-free and CCLAD systems provided lower pain scores without compromising hemodynamic stability. These results indicate that the optimal anesthetic approach is influenced not only by tissue mechanics but also by device characteristics and the patient's psychological profile. Tailoring device selection to individual stress responses may help redefine pain control strategies in dentistry and support a shift toward more personalized local anesthesia.

## Figures and Tables

**Table 1:** Table Pre- and post-injection fear scores and Δfear values (VAS 0–10) for each injection group.

Group	ΔFear (Mean±SD)	95% CI	Hedges'g vs. CDS	p-value*
CDS	-0.62±1.69	-1.14 to -0.10	0.00	Ref.
MCJ	-1.60±1.93	-2.20 to -1.00	-0.54	<0.05 vs. CDS
PCJ	-2.70±2.57	-3.50 to -1.90	-0.95	<0.001 vs. CDS
NFI	-1.15±1.55	-1.63 to -0.67	-0.32	n.s.
CCLAD	-1.65±2.13	-2.31 to -0.99	-0.53	<0.05 vs. CDS

Values are presented as mean±SD, with 95% confidence intervals and Hedges' g relative to CDS. Kruskal–Wallis test indicated a significant group effect (H=19.22, p=0.001). Post hoc pairwise comparisons showed significantly greater fear reduction with PCJ versus CDS and NFI (p<0.05), and with MCJ and CCLAD versus CDS.

**Table 2:** Table Post-injection pain scores (VAS 0–10) for each injection group.

Group	VAS Pain (Mean±SD)	95% CI	Hedges'g vs. CDS	p-value*
CDS	3.35±2.01	2.73 to 3.97	0.00	Ref.
MCJ	4.05±2.44	3.29 to 4.81	0.31	n.s.
PCJ	3.42±2.81	2.55 to 4.29	0.03	n.s.
NFI	3.17±1.82	2.61 to 3.73	-0.09	n.s.
CCLAD	3.14±1.77	2.59 to 3.69	-0.11	n.s.

Values are presented as mean±SD, with 95% confidence intervals and Hedges'g relative to CDS. Kruskal–Wallis test: No significant group effect (H=4.20, p=0.380). All post hoc pairwise comparisons were non-significant (p>0.05).

**Table 3:** Table Mean pulse rates (bpm) at baseline (T0), during injection (T1), and after injection (T2), and changes (ΔT1–T0, ΔT2–T1) across injection groups.

Group	ΔT1-T0 (Mean±SD)	95% CI	Hedges'g vs. CDS	ΔT2-T1 (Mean±SD)	95% CI	Hedges'g vs. CDS
CDS	1.6±4.3	0.27 to 2.93	0.00	-1.2±3.9	-2.41 to 0.01	0.00
MCJ	1.1±3.8	-0.08 to 2.28	-0.12	-1.2±3.7	-2.35 to -0.05	0.00
PCJ	0.7±3.5	-0.38 to 1.78	-0.23	-0.5±3.6	-1.62 to 0.62	0.18
NFI	0.9±3.6	-0.22 to 2.02	-0.17	-1.2±3.8	-2.38 to -0.02	0.00
CCLAD	2.9±4.8	1.41 to 4.39	0.28	-2.5±4.3	-3.83 to -1.17	-0.31

Values are presented as mean ± SD, with 95% confidence intervals and Hedges'g relative to CDS. Kruskal–Wallis tests indicated significant differences in ΔT1–T0 (H=19.16, p=0.001) and ΔT2–T1 (H=38.79, p CDS and MCJ for ΔT1–T0, and CCLAD < MCJ for ΔT2–T1 (p<0.05).

**Table 4:** Table Peripheral oxygen saturation (SpO2%) values at baseline (T0), during injection (T1), and after injection (T2), and changes (ΔT1–T0, ΔT2–T1) across injection groups.

Group	T0 (Mean±SD)	95% CI	T1 (Mean±SD)	95% CI	T2 (Mean±SD)	95% CI	ΔT1-T0 (Mean±SD)	Hedges'g vs. CDS	ΔT2-T1 (Mean±SD)	Hedges'g vs. CDS
CDS	98.1±0.7	97.9-98.3	98.2±0.6	98.0-98.4	98.2±0.7	98.0-98.4	+0.1±0.46	0.00	0.0±0.46	0.00
MCJ	98.3±0.6	98.1-98.5	98.4±0.7	98.2-98.6	98.3±0.6	98.1-98.5	+0.1±0.46	0.00	-0.1±0.46	-0.21
PCJ	98.2±0.8	97.9-98.5	98.3±0.7	98.1-98.5	98.4±0.6	98.2-98.6	+0.1±0.53	-0.00	+0.1±0.46	0.21
NFI	98.1±0.9	97.8-98.4	98.2±0.7	98.0-98.4	98.1±0.8	97.9-98.4	+0.1±0.57	0.00	-0.1±0.53	-0.20
CCLAD	97.9±0.8	97.7-98.2	97.8±0.9	97.5-98.1	98.0±0.7	97.8-98.2	-0.1±0.60	-0.37	+0.2±0.57	0.38

Values are presented as mean±SD, with 95% confidence intervals and Hedges'g relative to CDS. Kruskal–Wallis and Friedman tests indicated no significant intra- or intergroup differences (p>0.05). All values remained within normal physiological range; no desaturation or adverse events were observed.

## Data Availability

Declared none.
